# Recollections of a Rebel Surgeon, and Other Sketches; or, in the Doctor’s “Sappy” Days

**Published:** 1899-10

**Authors:** 


### TEXAS MEDICAL JOURNAL.

AUSTIN, TEXAS.

A MONTHLY JOURNAL OF MEDICINE AND SURGERY.

F. E. DANIEL, M. D., Editor.

Published Monthly at Austin, Texas, by Drs. Daniel and Hudson. Subscription price $1.00 a year in advance.

Eastern Representative: John Guy Monihan, St. Paul Building, 220 Broadway, New York City.

Official organ of the West Texas Medical Association, the Houston District Medical Association, the Austin District Medical Society, the Brazos Valley Medical Association, the Galveston County Medical Society, and several others.

### RECOLLECTIONS OF A REBEL SURGEON, AND OTHER SKETCHES; OR, IN THE DOCTOR'S "SAPPY" DAYS.

☞ Dr. Daniel's book which was announced some time ago ready for delivery next month. It is a series of short sketches, under the above title as forthcoming, is now in press, and will be mostly personal reminiscences of army life in camp, field and hospital during the War of Secession. It deals very little with the professional duties of an army surgeon, but is, for the most part, recollections of humorous occurrences, and is written pretty much in the vernacular of the South, in a free and unconventional style, and it is hoped will be read with interest by both lay and professional men. It will be gotten out here in Austin by the Von Boeckmann-Schutze Publishing Company, the printers of the *Journal,* in handsome linen, embossed covers, copiously illustrated with drawings by Austin artists, and will be sold at $1.00 a copy, by *subscription only.* It will be a "good thing" for our exchanges to offer as a premium for new subscribers, and the writer hopes they will send in their orders promptly and make sure of what they want, as the edition will be small, and will be taken up mostly in Texas. Individual subscribers are requested to send in their orders, with $1.00 (postage 10c. extra), without delay, and thus secure a copy.

To publishers who wish lots for premiums a discount of ten per cent. will be made. Address orders to Dr. Daniel. Exchanges who have a kindly interest in the Red-Back or the editor will greatly oblige the author by giving this a send-off. See advertising pages for announcement, contents of book and "opinions of the press." ☞

