# Erratum

**Published:** 1899-11

**Authors:** 


**Erratum.**—In the October number of the Journal the second and third lines in the editorial announcement of Dr. Daniel's book got transposed in making up the form, after proof had been revised, thereby getting the meaning a little mixed.

